# CrustyBase: an interactive online database for crustacean transcriptomes

**DOI:** 10.1186/s12864-020-07063-2

**Published:** 2020-09-14

**Authors:** Cameron J. Hyde, Quinn P. Fitzgibbon, Abigail Elizur, Gregory G. Smith, Tomer Ventura

**Affiliations:** 1 Genecology Research Centre, University of the Sunshine Coast, Sippy Downs, Queensland 4556 Australia; 2 Institute for Marine & Antarctic Studies (IMAS), University of Tasmania, Private Bag 49, Hobart, TAS 7001 Australia

**Keywords:** RNA-seq, Crab, Lobster, Shrimp, Crayfish, Gene, Expression, Search, Visualize, Genomics

## Abstract

Transcriptome sequencing has opened the field of genomics to a wide variety of researchers, owing to its efficiency, applicability across species and ability to quantify gene expression. The resulting datasets are a rich source of information that can be mined for many years into the future, with each dataset providing a unique angle on a specific context in biology. Maintaining accessibility to this accumulation of data presents quite a challenge for researchers.

The primary focus of conventional genomics databases is the storage, navigation and interpretation of sequence data, which is typically classified down to the level of a species or individual. The addition of expression data adds a new dimension to this paradigm – the sampling context. Does gene expression describe different tissues, a temporal distribution or an experimental treatment? These data not only describe an individual, but the biological context surrounding that individual. The structure and utility of a transcriptome database must therefore reflect these attributes. We present an online database which has been designed to maximise the accessibility of crustacean transcriptome data by providing intuitive navigation within and between datasets and instant visualization of gene expression and protein structure.

The site is accessible at https://crustybase.org and currently holds 10 datasets from a range of crustacean species. It also allows for upload of novel transcriptome datasets through a simple web interface, allowing the research community to contribute their own data to a pool of shared knowledge.

## Background

In recent years, the advancement of next-generation sequencing (NGS) technologies have provided new and exciting opportunities for biologists in a variety of disciplines. A distinguishing feature of NGS technologies is that they can be applied with relatively few taxonomic limitations, thereby extending their utility well beyond model species such as the fruit fly *Drosophila melanogaster* or the mouse *Mus musculus*. While the latter species provide us with an increasing depth of knowledge in the fields of genetics and genomics, NGS technologies provide an opportunity to expand and contextualize our theories and hypotheses in a variety of taxonomic and biological settings [1]. However, a lack of appropriate infrastructure presents a significant bottleneck in taking advantage of this breadth of information.

Total RNA sequencing, commonly known as RNA-seq or transcriptome sequencing, has been an effective tool for curating and characterising genes across an expanding range of species in the past five years. This is well-reflected in gene expression data repositories held by the National Centre for Biotechnology Information (NCBI) [2], where the sequencing-based experiments hold over nine-times the species diversity than that of array-based experiments.

RNA-seq analysis results in two fundamental data types corresponding to each mRNA transcript in the sample:

1. **Nucleotide sequence data** based on alignment and assembly of sequencing reads.
2. **Expression data** based on the number of reads which align to each transcript sequence.

The combination of these data types is what gives RNA-seq such unprecedented power. A de novo transcriptome provides many of the insights associated with conventional gene sequencing, such as single-nucleotide polymorphism (SNP) detection, protein structural analysis and comparative evolutionary analysis. A typical RNA-seq pipeline further augments these data by matching transcript sequences to known genes to provide “annotations” which can then be directly queried by keyword search. But the addition of accurate gene expression quantitation provides a new dimension to these datasets by showing how these sequences are expressed in a particular environment, tissue or point in time [3]. These traits of RNA-seq data have been well-utilized in aquaculture research, where the industry depends on expanding knowledge in a wide range of species with little previous genomics knowledge [4].

Since genomics data is conventionally limited to describing sequence data, the main criteria when searching available data is taxonomy or genotype. As such, these data need only to be indexed by the species or individual to which they relate in order to be found by a user. However, the presence of gene expression data in RNA-seq adds a new dimension to these datasets. Here, the researcher is concerned not only with the identity of the subject, but also the conditions from which the data were derived. For example, a researcher concerned with neurological development in decapods may be delighted to find a time-series RNA-seq dataset describing juvenile brain ontogeny in any crustacean species. The additional context behind these datasets requires special consideration to ensure that they are accessible to researchers.

With this in mind, it is important to make the distinction between data availability and accessibility. While a specific stream of data may be broadly available online, whether or not that data is actually utilized depends strongly on accessibility. If a researcher can only access one dataset per hour, then the data is far less accessible than if they could access 1000 datasets per hour. Pragmatically speaking, access to online data is typically limited by the searchability of the data and the format in which it is then presented. Such limits on accessibility impose an obvious barrier to the dissemination of information and are of utmost importance if public data accessibility is to be taken seriously.

While much of the world’s RNA-seq data is publicly available (as required by many funding institutions and journals), we believe that accessibility to this data is far from optimal. Current platforms are well-equipped for sharing sequence data, and indeed many transcriptome sequence archives held by the NCBI can be queried directly with their freely-available Basic Local Alignment Search Tool (BLAST), a resource that has become entirely ubiquitous in the bioinformatics sphere. This incredibly efficient tool allows millions of nucleotide or protein sequences to be searched within seconds by matching them against a query sequence provided by the user [5].

However, a conventional sequence-oriented platform is far from adequate when it comes to accessing expression data; the best solution offered by NCBI allows researchers to upload spreadsheets of expression data as supplementary files to a corresponding Gene Expression Omnibus (GEO) record. As a result, the absolute minimum effort required to utilize this data is to download these files, create a local BLAST database and then manually cross-examine BLAST results from within a spreadsheet. In the majority of cases these files are not available, and one might need to resort to full assembly and read-mapping from raw sequencing reads which could take several weeks. This forms a significant barrier to the dissemination of public data in two ways; first by being incredibly time-consuming, and second by limiting access to only those with sufficient bioinformatics expertise and computational resources. Despite the widespread public availability of these datasets (71,818 NCBI bioproject records as of 01/11/2019), the accessibility of these data remains limited.

Outside of the NCBI platform, it has become conventional for research groups to package the NCBI’s BLAST toolkit for the purpose of sharing genomics data in a dedicated online environment. SalmoBase [2, 6] is an online platform for sharing genomics and transcriptomic data of salmonid species, incorporating the BLAST toolkit and GBrowse genome browser framework [7], as well as keyword searching for annotated genes and transcripts. This formula is quite characteristic of online genomic platforms, which present data from various collections of species, from nematodes [8, 9] and echinoderms [2, 10] to human pathogens [2, 11]. These platforms generally prioritise the navigation and visualisation of sequence data, with gene expression information making an occasional appearance. Recently we have seen the release of the Crustacean Annotated Transcriptome (CAT) database, a platform with similar structure to the aforementioned databases and populated with transcriptomes of seven crustacean species [2, 12]. This database makes a notable effort to represent expression data by integrating a differential expression analysis tool [13] for three of these species. In a showcase for what is possible with more liberal investment, the Allen Brain Map [2, 14] provides a three-dimensional interface for viewing gene expression in the human brain. While these applications offer an improvement on data accessibility, they are unfortunately not designed to represent data for the wider research community.

Despite the abundance of these online databases, we have yet to see a platform which might permit access to the breadth of transcriptomic data available to the genomics community. Indeed, one of the most important attributes of a database is that it should seek to bring data together into one easily-accessible location, since it is far easier to search a single location than to navigate many different sources [15]. We present here a platform which aims to reconcile RNA-seq datasets through community engagement. This platform combines familiar search tools with an intuitive graphical interface to provide simple and effective visualization of gene expression data. The design and development of this RNA-seq platform was motivated by three key goals:

1. Efficient navigation between datasets
2. High accessibility of gene expression data within datasets
3. Scalable across species and experimental designs

This platform is comprised of three core features. We leverage the ubiquity of the BLAST tool as a means for searching and accessing transcript sequences, whose corresponding expression data are instantly rendered in an interactive graphical output. We also provide an interface for navigating the datasets themselves, allowing the user to search not only the species, but also the biological context of RNA-seq experiments. In order to utilize the scalability of this platform, we have also implemented a data import pipeline which allows users to upload new RNA-seq datasets through the web interface. These three core features result in a platform which can grow organically, while providing researchers with streamlined access to the variety of taxonomic and molecular insights that they collectively produce. In consideration of present funding and logistical limitations, however, we have restricted the scope of this platform to crustacean species. Therefore, in acknowledgement to the apparent naming convention of genomics databases, this platform has been released under the name “CrustyBase”, and is accessible at https://crustybase.org. The site currently holds ten public datasets obtained from NCBI Gene Expression Omnibus records and from our own archive, including species such as the ornate spiny lobster *Panulirus ornatus* {Hyde, 2019 #442}, the Eastern spiny lobster *Sagmariasus verreauxi* {Ventura, 2015 #139;Ventura, 2015 #64}, the tropical land crab *Gecarcinus lateralis* [16], the whiteleg shrimp *Litopenaeus vannamei* [17, 18], the oriental river prawn *Macrobrachium nipponense* [19], the marine copepod *Temora longicornis* [20], the salmon louse *Caligus rogercresseyi* [21] and the water flea *Daphnia magna* [22].

## Construction and content

**Fig. 1:**
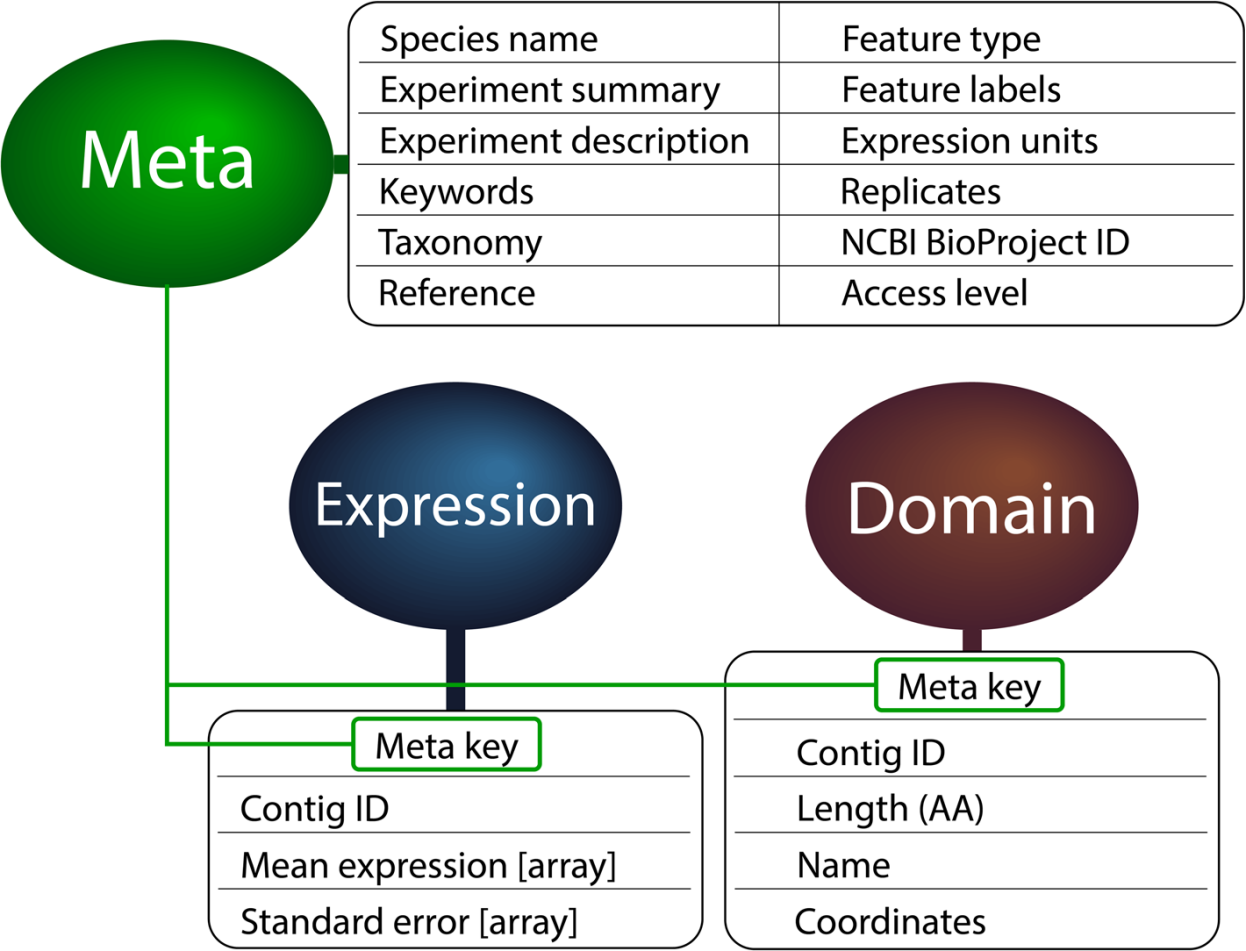
Data model architecture. The Meta, Expression and Domain models define the core of the database schema, with each Meta entry serving as a master record for each dataset. Each Meta record is linked to associated Expression and Domain records through a one-to-many relationship (shown by green lines and boxes). Adjacent tables show the main attributes of each model. “Features” describe the experimental variables used in the study, such as tissue type, treatment or phenotype

CrustyBase (CB) comprises a backend programmed in Python 3.7 and built on the Django 2.1 web framework with a PostgreSQL database. All software used in construction is open-source and freely-available. The Django web framework includes a variety of features designed to accelerate web development, including database integration and a built-in admin application and user authentication system. A Django application builds on top of these features by integrating original, self-contained apps which perform the various functions that are required by the website. HTTP responses are dynamically rendered from HTML templates, with CSS and JavaScript for interface styling and logic in the frontend (i.e. performed within the user’s web browser). To enhance aesthetics and functionality of CB several open-source CSS and JavaScript libraries have been utilized such as Bootstrap 4 for user interface styling, Two.js for rendering protein domain plots and Plotly.js for rendering transcript expression graphs. Pre-computed data from each RNA-seq experiment are represented by three Django models: Meta, Expression and Domain. Django stores these models as tables in the PostgreSQL database. The Meta model serves as a root for each dataset and stores various metadata relating to the experiment such as the organism name, taxonomic information, institution of origin and experiment description (Fig. 1). The Meta model is key to enabling data accessibility, as it allows datasets to be queried across all of these fields. Once the Meta record has been fetched for an experiment, the associated Expression and Domain objects linked to that record can be obtained through the one-to-many relationship. The Expression model corresponds to a single transcript, describing the mean and standard deviation of transcript expression across the experimental features (i.e. tissue, developmental stage, treatment). The Domain model corresponds to a single protein domain and may return many records for a particular transcript. Each record describes the transcript ID, name, peptide coordinates and accession number of a predicted protein domain.

In order to allow uploading and importing of new RNA-seq data by CB users, we have implemented a web interface which gathers the required files and information from the user. To ensure data integrity and formatting, uploaded files are validated on the webserver. These files are then sent to a remote data server by Secure File Transfer Protocol (SFTP). After processing, the completed data is returned to the web server by SFTP (in the case of FASTA files and BLAST databases) and remote import to SQL database (in the case of expression and domain data), at which point they become available to users of the website. The import pipeline implemented on the data server is written in Python 3.6.5 and utilizes the TransDecoder program [23] for proteome prediction and a local build of the NCBI’s CD-Search tool [24], which uses RPS-BLAST to match protein sequences against the CDD database of conserved protein domains (obtained from ftp://ftp.ncbi.nih.gov/pub/mmdb/cdd). Other tasks carried out by the pipeline include calculation of mean and standard error from transcript expression data and formatting of data in preparation for database upload. A detailed outline of this import pipeline can be found in supplementary file 1.

**Fig. 2:**
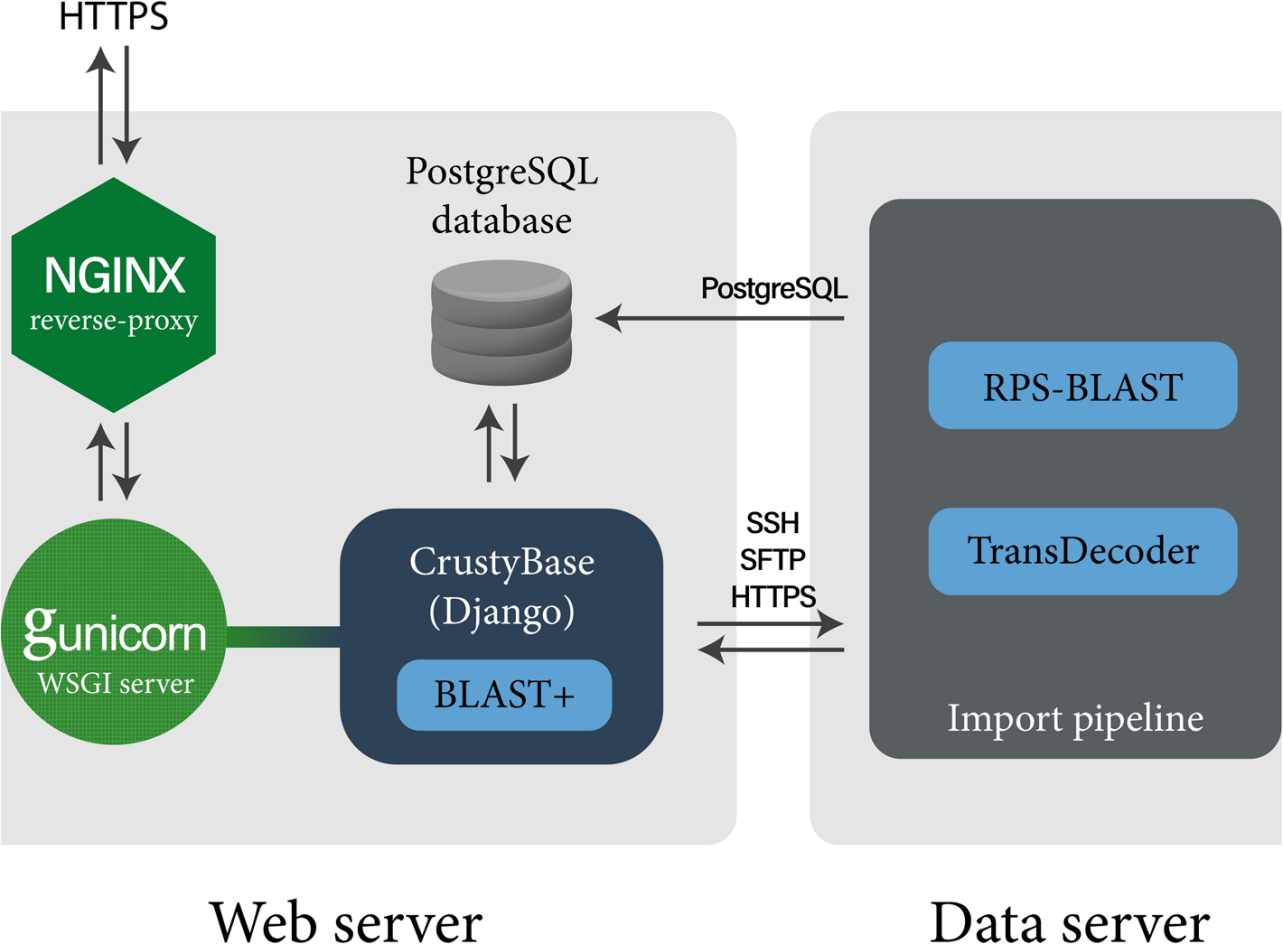
Server architecture. Nginx functions as a reverse proxy, handling HTTP requests and passing them on to Gunicorn which then distributes them to a worker thread. These worker threads then execute request handlers defined in the CrustyBase codebase. RNA-seq datasets uploaded for import are routed to a dedicated data server for processing. New datasets are remotely imported to the PostgreSQL database when processing is complete

The CB web server is a virtual machine provisioned by the National eResearch Collaboration Tools and Resources project (Nectar). The server runs Ubuntu 18.04 with web-serving performed by Gunicorn [25] through a reverse-proxy with Nginx [26], as depicted in Fig. 2. The data server is a high-performance computer running Ubuntu 16.10.

CB was originally populated, tested and developed with a dataset from the ornate spiny lobster *Panulirus ornatus*. This time-series transcriptome spans twelve stages of larval development, describing gene expression from the late phyllosoma, through the puerulus, to the juvenile lobster. During this time the animal goes through five molts, two of which involve a metamorphosis. This dataset was used to curate the nuclear receptor gene family and describe their activity throughout these developmental events [27]. We consider this a pilot dataset for CB and it will remain in the database with full public access (access levels are described in the Utility section).

### Utility

The functionality of CB is broken down into distinct apps. Two apps perform the majority CB’s utility. The BLAST tool allows users to search an RNA-seq dataset in the conventional manner, with expression and predicted domain data being instantly accessible in the form of graphs and figures. The data browser helps users to explore datasets by searching not only by species, but also by experimental conditions and attributes. This allows researchers to find datasets which are relevant to a specific interest (for example molting) that transcends taxonomic boundaries.

The BLAST tool presents users with familiar input fields – a text box for query sequence entry, a list of databases to search and a choice of BLAST algorithms to allow either protein or nucleotide queries. There are more parameters that a user might wish to specify when making a BLAST search, and we may incorporate more of these parameters into the user interface in future. When the BLAST search is complete the user is presented with a “stack” of result panes, each pertaining to one of the selected datasets. Each pane shows a summary table of transcripts ordered by match score (this will be familiar to many BLAST users) accompanied by a generic image of the subject species and a brief description of the experiment details. The user can scroll down this page to get a brief overview of the BLAST hits across the selected datasets. When a user is interested in a particular dataset’s results, they can choose to “expand” the result view, thereby zooming in and filling the screen with the selected dataset. This detailed view including a BLAST alignment, transcript expression graph and protein structure plot, which update instantly as the user cycles through the matching transcripts with either arrow keys or mouse clicks. This provides users with immediate insights into the bioactivity and structure of the selected transcripts, with several consequences. Firstly, it allows more accurate identification of a biologically relevant transcript; the highest-scoring match might in fact be a transcript with very low expression or a truncated protein structure. Secondly, discrepancies in gene activity are immediately brought to the user’s attention, making it possible to browse and compare the expression of genes between available datasets.

The data browser is composed of two interfaces which are designed with simplicity in mind. The first view presents the user with a list of all available datasets. Each dataset is represented by an image of the animal, species name, number of replicates and a brief description of the RNA-seq experiment. At the top of the page is a single text input field which can be used filter the datasets shown in real time by entering keywords relevant to the user. These keywords could be a scientific or common name, taxonomy, or biological keywords such as “molt”, “brain” or “immune”. The user can then scroll down the page and select a dataset of interest, bringing them to the second interface of the data browser. This page provides a detailed view of the selected dataset, including the dataset owner, assembly statistics, institution, reference and descriptions of the species, experiment and assembly procedure. From either of these pages the user can jump directly to the BLAST search tool with the database selected.

Users are given the opportunity to import RNA-seq datasets of their own through the CB web interface through a carefully designed import dialog. This requires that users are logged in to CB and are a member of a Group. Groups are designed to manage ownership of datasets in a manner that reflects data creation and ownership in the real world, and helps researchers share access of datasets with colleagues and collaborators. Any user can create a group, request to join a group or invite users to join a group. A user can be a member of more than one group. When a user uploads a dataset, ownership is delegated to one of the user’s groups. This has several important considerations: 1) Every user in the group has full access to the data. 2) If a user account is deleted, all data uploaded by that user remains in the group. 3) Any member of a group can delete and modify datasets owned by that group. 4) If all members leave a group, the group is deleted along with all datasets under that group’s ownership. However, we are aware that groups become redundant for datasets which are already in the public domain. In this case, the user can choose to omit group delegation and simply import the dataset into the public domain. This makes the dataset fully accessible to all users by default, and streamlines the import of public datasets.

After delegating a group for data ownership, the user fills out a form which describes all meta data relevant to the data set, such as species name and experimental conditions. These fields are essential to effectively finding and displaying the dataset. The user can then choose whether the dataset will have full or partial public accessibility. Full accessibility allows any CB user to download raw sequence and expression data, while a dataset with partial accessibility only provides public CB users with a graphical view of the data. The principle behind this design is to incentivise researchers to share data which might otherwise remain private – we hope that users who find interesting results in restricted datasets will seek collaboration with the dataset owners.

After meta data has been entered and accepted the user has to upload two files: a FASTA-formatted sequence file which contains the transcriptome assembly, and a CSV-formatted spreadsheet which contains the expression data for each transcript. These files will be parsed and tested for integrity, then returned to the user if any errors are encountered (i.e. missing data, incorrect data or mismatching contig identifiers between sequence and expression data). If these files pass validation, the user is presented with a review page where they can check that their data has been correctly interpreted. The user then has the option to make revisions to the import before final submission. We expect that a dataset should become available on CB within 48 h after import.

There are several further utilities that we hope to incorporate into CB in the future in order to enhance the utility of this resource for the research community. These additions aim to improve user access to the database, introduce new data types to add value to datasets, and streamline the ingestion of new datasets into the database. Our development proposal for the future is outlined below, but we also welcome feedback from the community either by email or through the feedback form on the CB website.

### Priorities for the next major release of CrustyBase

1. Search and display transcripts by protein domains. This feature would allow users to keyword-search for protein domains, and then view all transcripts in a dataset which are predicted to encode the selected domain(s).

### Long-term additions and improvements

1. Implement transcript annotation in the data import pipeline. This would allow users to search for transcripts by gene name, as well as view annotation information for any transcript that they find.
2. Transcriptome assembly and quantitation in the data import pipeline. This would allow direct import from raw sequencing reads and would also standardise the quality of assemblies across the database. However, this would require an in-depth feasibility study as it is unclear whether this would scale across datasets of different size and quality.
3. Provided the above, enable direct import of datasets from NCBI BioProject/sequencing read archive (SRA). This would enable CB to ingest a large quantity of publicly available data held in the SRA, which holds 5629 sequencing runs from crustacean RNA-seq projects as of 18/02/2020.

### Use case

In order to demonstrate the utility of CB to prospective users we will run through a brief use case to demonstrate the manner in which data can be accessed and retrieved in various formats. To investigate the possibility that the developmental gene *sonic* is conserved in crustacean lineage, we will examine a basic set of research questions:

1. **Do crustaceans possess an ortholog for the Sonic Hedgehog gene?**
2. **If so, what is its activity throughout larval development?**

**Fig. 3:**
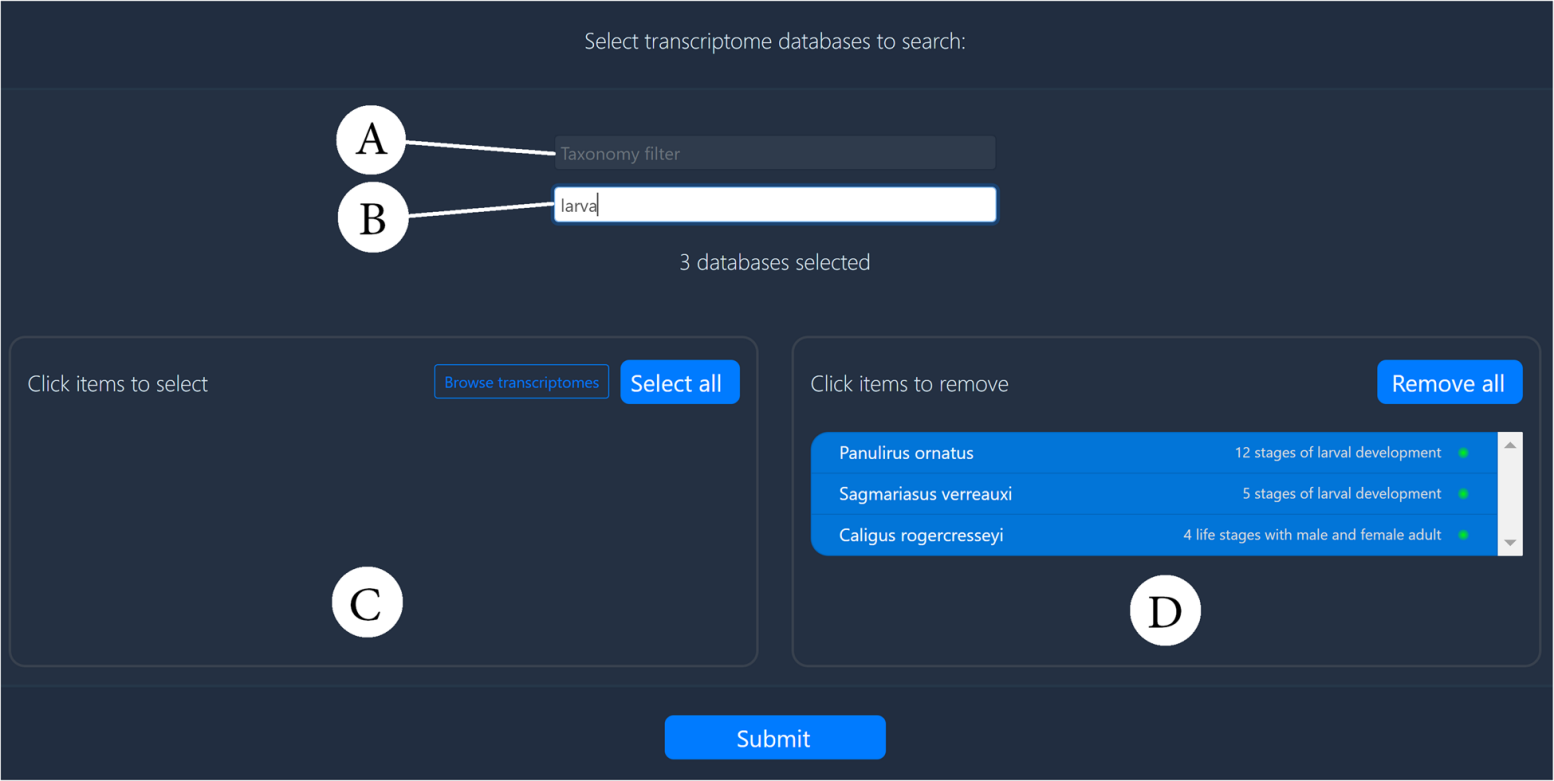
BLAST database selection. Database selection in the BLAST interface allows for trivial searching of datasets by two text-based input filters (**a** and **b**). Input A allows for filtering based on the available taxonomy in the CB database on the basis of class, order, genus and species. Input B allows for keyword filtering of experimental conditions. In this case, the term “larva” filtered the available datasets (**c**) to three transcriptomes, which could then be added to the selection (**d**) in a single click

To begin with, we need a query sequence for *sonic* to have any hope of finding it somewhere in CB. A quick search in the NCBI protein database with the keywords “Sonic SHH” yields a single protein sequence of 126AA belonging to the barnacle *Amphibalanus amphitrite* (accession KAF0307803.1)*.* We copy the FASTA formatted sequence for this protein and jump over to CB’s BLAST tool at https://crustybase.org/blast, where we paste the sequence into the query input and select TBLASTN as our search algorithm, to permit a protein query. We can then consider which transcriptome datasets should be searched, considering our question regarding larval development. We type into the keyword filter “larva” to find three related datasets (two spiny lobsters and one salmon louse), which we add to the “selected” pane (Fig. 3) before hitting the “submit” button.

**Fig. 4:**
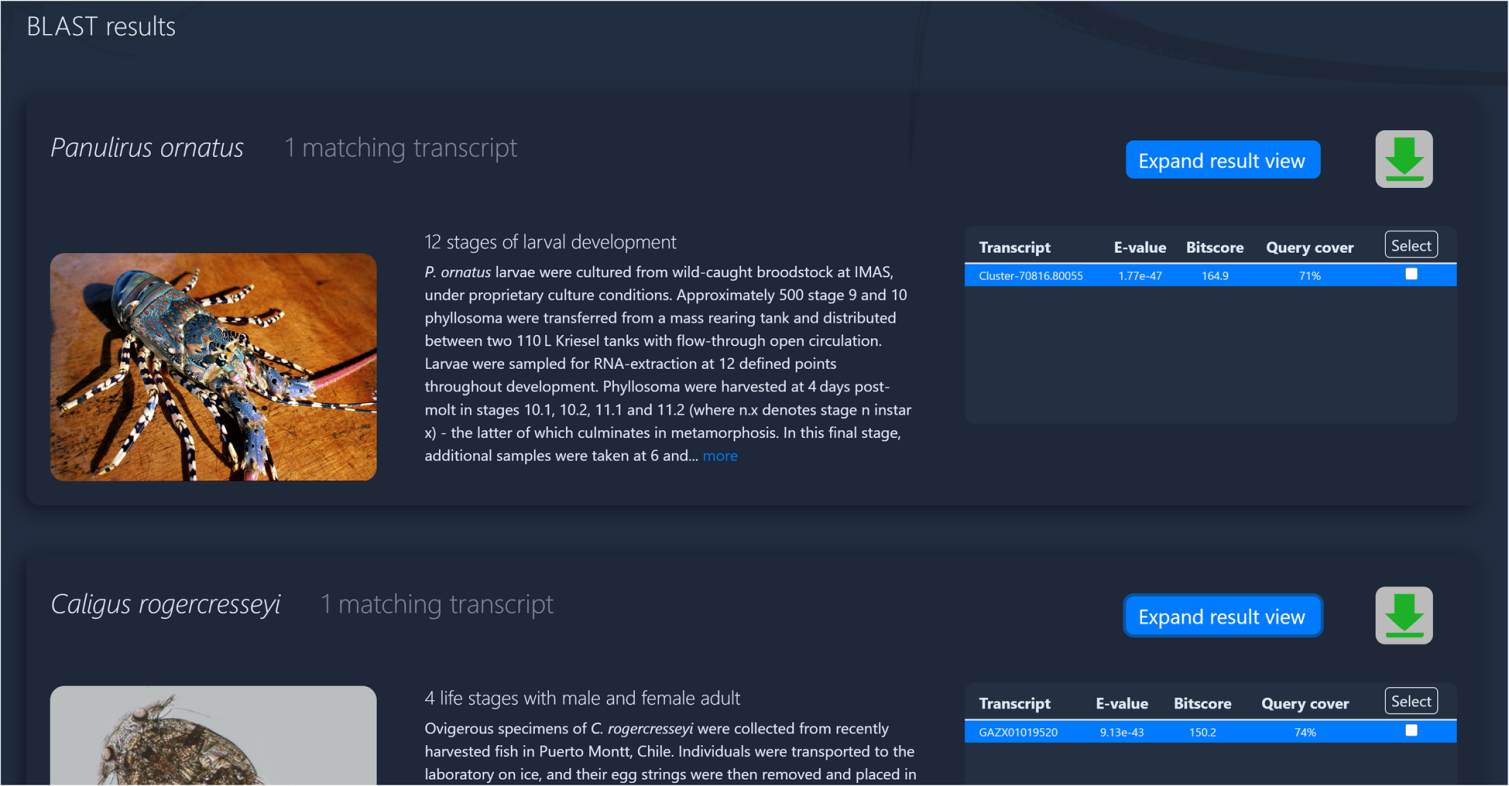
BLAST results – overview. Target datasets appear as a stack of “panes” showing a generic image of the animal with a brief description of the experiment and a hit table of BLAST summary statistics. Here, the user can scroll down the page to get an overview of all datasets with transcripts matching their query sequence. The user can then click the “expand” button (top-right) for a detailed view of that dataset, or select transcripts for data download (top-right)

**Fig. 5:**
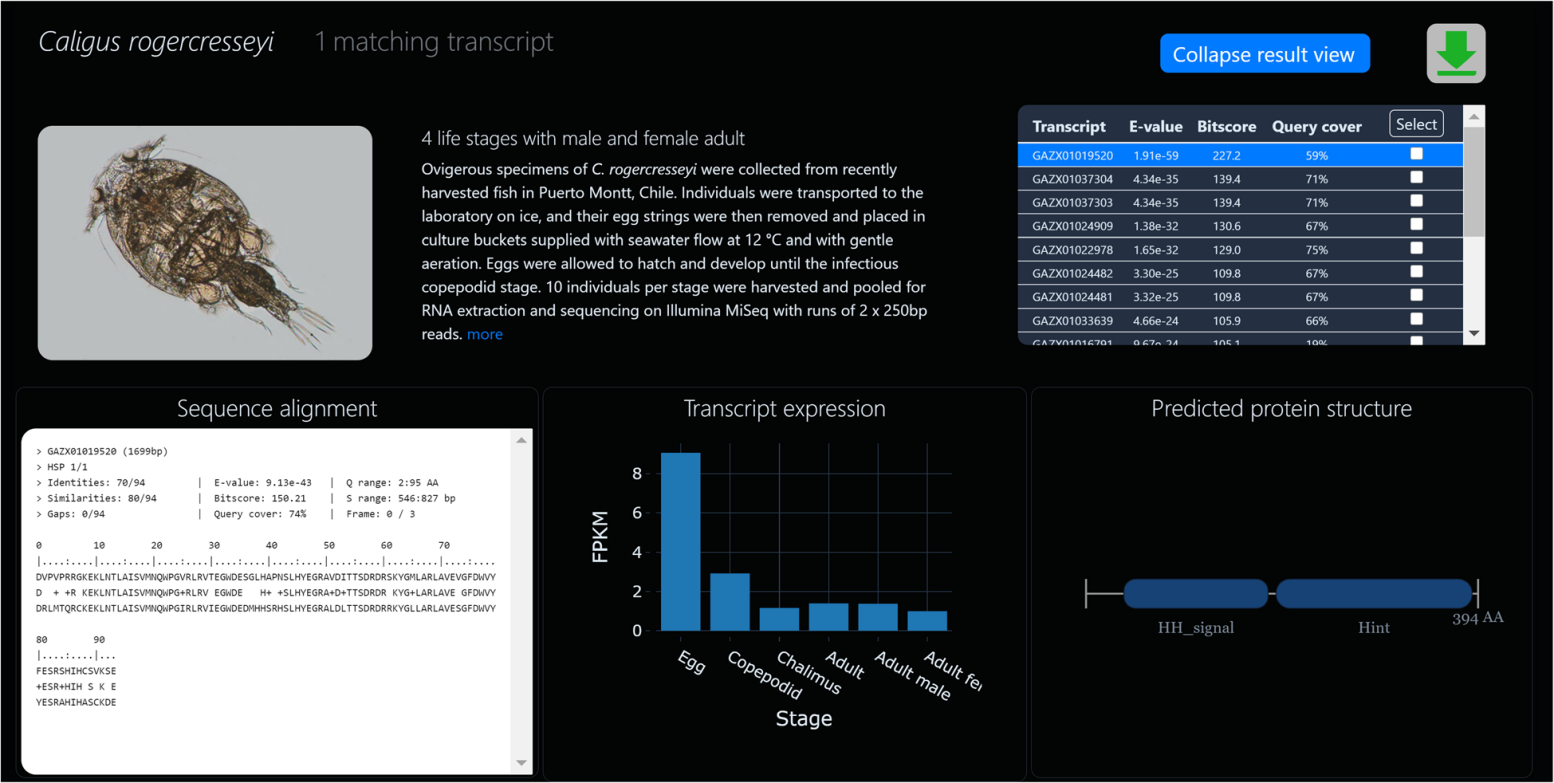
BLAST results - expanded view. After expanding a dataset, the user receives a full-page, detailed view of that dataset’s BLAST hits including the BLAST alignment and statistics (bottom-left), an interactive expression graph (bottom-middle), and a predicted protein plot (bottom-right). The hit table (top-right) shows summary statistics for matching transcripts. The user can navigate these hits with either arrow keys or mouse clicks while the below panes update in real-time to display data for the selected transcript

Eight seconds later we find a single match for s*onic* in both *Panulirus ornatus* and *Caligus rogercresseyi* (Fig. 4). Both have matched with quite a moderate E-value of around 10^− 45^, but after expanding these datasets we can see from the BLAST alignments that they are quite a good match with around 90% identity (Fig. 5). Both species show sufficient expression to suggest bioactivity in these transcripts (180 RLE and 9 FPKM, respectively). Expression levels indicate that *sonic* activity does indeed vary according to developmental stage (Fig. 5). In the salmon louse, we see three-fold upregulation in the egg. In the spiny lobster we see 2-fold higher expression before the phyllosoma metamorphosis, extending well into the puerulus phase.

Looking at the protein prediction, we being to see a distinction between these two transcripts. The lobster *sonic* appears to encode a protein of only 69AA, with no predicted domains. At half the length of our query, this looks like a truncated protein. The salmon louse *sonic*, on the other hand, encodes a much larger protein of 394AA (Fig. 5). Two predicted domains confirm its identity beyond much doubt: “Hint” (Hedgehog/Intein) and “Hedgehog amino-terminal signalling domain”. So, it appears that our barnacle query sequence was in fact truncated, and we are perhaps now looking at the first full-length Sonic protein to be reported in a crustacean.

**Fig. 6:**
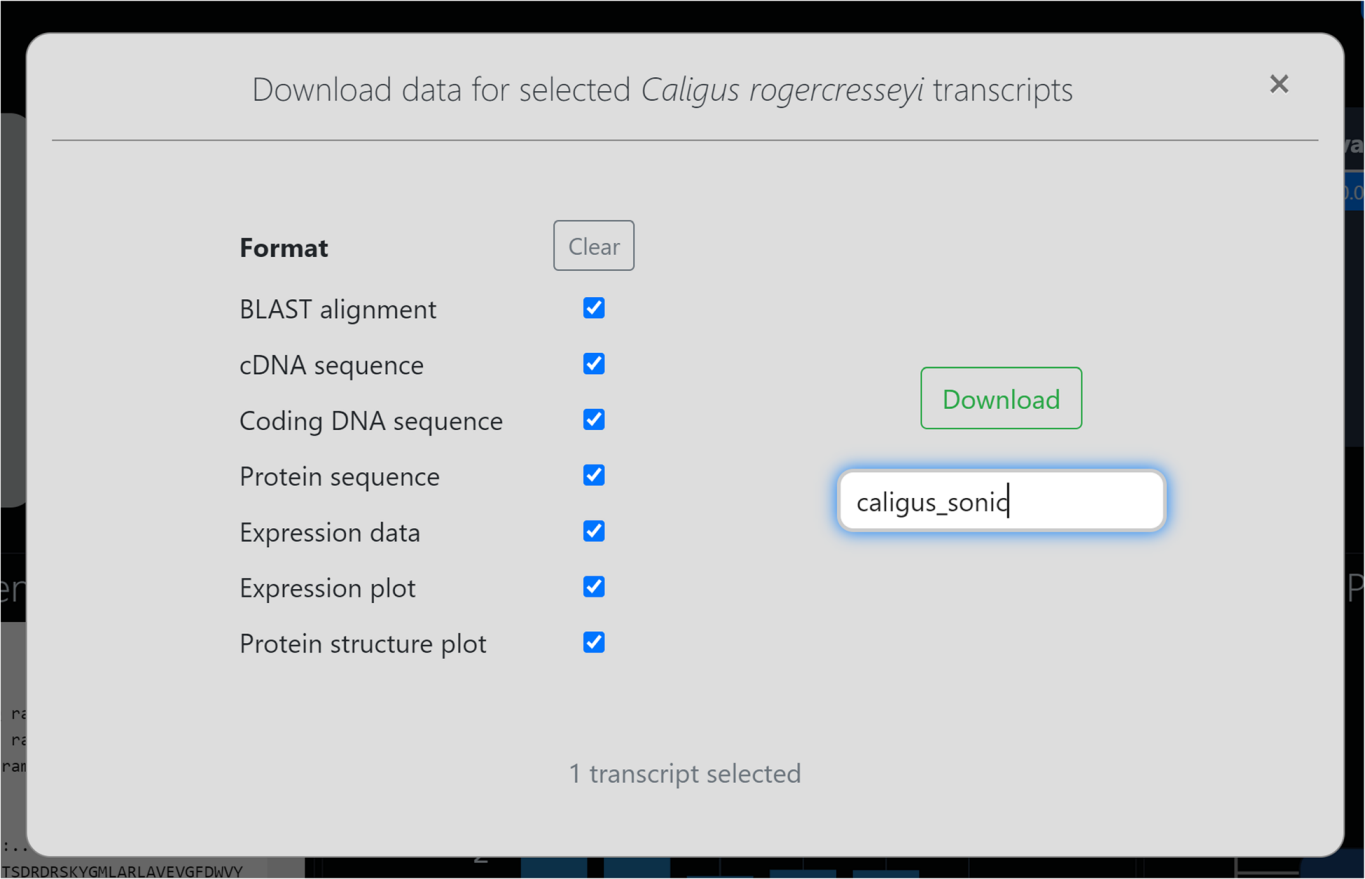
BLAST results – data download. This window appears when the user has selected one or more transcripts in the hit table (Fig. 4) and hit the download icon. The user can select the data formats most appropriate to them and enter a prefix for the pending file before downloading

Content with our findings, we now wish to secure some data for this interesting transcript. We select the checkbox for our transcript (Fig. 4), and hit the download icon above. It won’t take long to render data for a single transcript, so we may as well select all data types (Fig. 6). To ensure that we can remember the origin of this file in the future, we enter the file prefix “caligus_sonic” before downloading (Fig. 6). Two seconds later we have the file “caligus_sonic.zip” on our computer, containing our transcript’s DNA and protein sequences, expression data and graph, BLAST alignment and protein structure plot. With these data for future reference, we could begin a phylogenetic study by curating *sonic* transcripts from other datasets in CB, or jump back to NCBI to search for novel transcripts in the TSA archive.

Of course, this investigation could alternatively have been carried out with an NCBI BLAST search of the TSA archives, linking to related BioProject and GEO datasets, downloading the expression data as a spreadsheet and plotting it manually. To get our protein structures we would then have to return to NCBI to use the CD-search tool. But with the interface provided by CB, this entire process can be resolved within two web pages and around 10 min of the user’s time.

## Conclusion

In the genomics era, sharing and accessibility of biological data are of utmost importance. Much of the progress in this field can be attributed to the model organisms such as *Drosophila* and *Mus musculus* which have each attracted the shared attention of a large, well-funded research community. With the advent of NGS, however, even a moderately-equipped researcher has the ability to produce large, complex datasets. While these datasets may be valuable to the individual researcher who created them, they are an even greater asset when the community can unite their efforts to form a shared pool of information. Although CB was only designed to fulfil this purpose for a defined group of organisms, we hope that it can illuminate the potential for modern information technology and open-source software to solve these issues for the wider research community.

## Supplementary information

**Additional file 1.**

## Data Availability

CrustyBase is freely available to anyone with internet access. Access to raw sequencing data can be found through the corresponding NCBI BioProject where the creator of the dataset has provided this information. We strongly encourage that future contributors to CB make their data available in this way as this greatly enhances the credibility and utility of the data.
