# Supplementary material for: CrustyBase: an interactive online database for crustacean transcriptomes
Source: BMC Genomics. 2020 Sep 14;21:637. doi: 10.1186/s12864-020-07063-2 (PMC7490944; doi:10.1186/s12864-020-07063-2)
Supplement: Supplementary file 1 — Additional file 1. [file 12864_2020_7063_MOESM1_ESM.pdf]

Data import pipeline

1. **Preformat Ids (Reformat contig IDs if longer than 25 characters)**
2. **Format Meta data**
3. **TransDecoder.LongOrfs**

   ```
   -m 50
   ```

4. **TransDecoder.Predict**
5. **Parse TDC output to standardised FASTA format (formatting Ids mainly)**
   1. Protein FASTA
   2. CDS FASTA

6. **Predict domains:**
   1. TDC proteome broken into chunks of 5000 sequences
   2. Chunks processed in parallel (20 cpus with Python multiprocessing):

      `rpsblast` (NCBI BLAST+ toolkit)

      ```
      -query        <chunk-n>
      -evalue       0.01
      -seq          no
      -outfmt       5 (XML)
      -db           Cdd (ftp://ftp.ncbi.nih.gov/pub/mmdb/cdd)
      -out          <chunk-n.xml>
      ```

   3. XML files parsed to text format:

      `rpsbproc` (NCBI BLAST+ toolkit)

      ```
      -q
      -i            <chunk-n.xml>
      -o            <chunk-n.out>
      ```

   4. Text files parsed and combined to single CSV file (Python)

7. **Make blast database from assembly.fasta:**

   `makeblastdb` (NCBI BLAST+ toolkit)

   ```
   -in           <assembly.fasta>
   -out          <blast/assembly.fasta>
   -dbtype       nucl
   -parse_seqids
   ```

8. **Send FASTA files to webserver over SFTP**
9. **Parse and format expression and domain CSV files for bulk-import to webserver SQL database.**
10. **Notify webserver of import completion**
